# Сочетание первичного гиперпаратиреоза и саркоидоза у пацентки с гиперкальциемией

**DOI:** 10.14341/probl13550

**Published:** 2026-01-18

**Authors:** Л. И. Данилова, Г. Г. Короленко, М. Л. Лущик, И. И. Бурко, А. А. Романовский, С. В. Якубовский, О. Н. Исачкина

**Affiliations:** Белорусский государственный медицинский университетБеларусь; Belarusian State Medical UniversityBelarus; 10-я городская клиническая больницаБеларусь; 10th City Clinical HospitalBelarus

**Keywords:** ПТГ-зависимая и ПТГ-независимая гиперкальциемия, гиперкальциемический криз, саркоидоз, бисфосфонаты, PTH-dependent hypercalcemia, PTH-independent hypercalcemia, hypercalcemic crisis, sarcoidosis, bisphosphonates

## Abstract

Обсуждаемый клинический случай демонстрирует сложности ведения пациентов с впервые выявленной гиперкальциемией. С точки зрения вовлечения паратгормона (ПТГ) в механизмы развития гиперкальциемии выделяют паратгормонзависимую (ПТГ-зависимую) и паратгормоннезависимую (ПТГ-независимую) гиперкальциемию. В приведенном клиническом случае анализ клинических, лабораторных и инструментальных данных позволил выявить редкое сочетание ПТГ-зависимой и ПТГ-независимой гиперкальциемии. У пациентки Д., 74 года, был диагностирован первичный гиперпаратиреоз (ПГПТ) как причина тяжелой гиперкальциемии. Сохранение высоких значений кальция в сыворотке крови после проведенного хирургического лечения при достижении нормализации уровня ПТГ потребовало продолжения диагностического поиска и исключения иных причин гиперкальциемии. В результате был выявлен саркоидоз с поражением медиастинальных и шейных лимфатических узлов. После включения в схему терапии метилпреднизолона уровень кальция в сыворотке крови постепенно нормализовался.

## Abstract (en)

This clinical case demonstrates difficulties in managing patients with newly diagnosed hypercalcemia. Depending on the involvement of parathyroid hormone (PTG) in the mechanisms of hypercalcemia the latter is divided into parathyroid hormone-dependent (PTH-dependent) and parathyroid hormone-independent (PTH-independent). In this clinical case an analysis of clinical, laboratory and instrumental data revealed a rare combination of PTH-dependent and PTH-independent hypercalcemia. A 74-year-old patient was diagnosed with primary hyperparathyroidism (PHPT) which was the cause of severe hypercalcemia. Persistence of hypercalcemia after surgical treatment and normalisation of PTH levels required further diagnostic search and exclusion of other causes of hypercalcemia. As a result, sarcoidosis with lesions of the mediastinal and cervical lymph nodes was revealed. Methylprednisolone was administered in the treatment regimen, which gradually normalised serum calcium levels.

## АКТУАЛЬНОСТЬ

Кальций играет важную роль во многих физиологических процессах. Именно по этой причине уровень кальция в сыворотке крови поддерживается строго в узком диапазоне за счет регулирующего влияния паратгормона (ПТГ) на мобилизацию кальция из костей, а также на его всасывание в кишечнике и реабсорбцию в почечных канальцах посредством активации витамина D в почках. Гиперкальциемия является относительно распространенным клиническим состоянием. Выявляемость гиперкальциемии различается между странами из-за различий в подходах к диагностике и скринингу. По данным Walker M.D. и соавт., повышенный уровень кальция в сыворотке крови встречается примерно у 1% населения в целом [1].

Причины, вызывающие гиперкальциемию, многочисленны. У пациентов, госпитализированных по экстренным показаниям, первое место среди причин повышения сывороточного уровня кальция занимают злокачественные новообразования, гемобластозы (миеломная болезнь, лимфомы, лимфогранулематоз, лейкозы), составляющие, по данным разных авторов, 60–90% [2]. На долю первичного гиперпаратиреоза (ПГПТ) у данной категории пациентов приходится 10–40% [2][3][4]. В то же время, согласно результатам эпидемиологического исследования, проведенного в штате Калифорния, среди всех причин хотя бы единожды зарегистрированной гиперкальциемии у не госпитализированных лиц доля ПГПТ составляет 87% [5].

С точки зрения вовлечения ПТГ в механизмы развития гиперкальциемии ее делят на паратгормонзависимую (ПТГ-зависимую) и паратгормоннезависимую (ПТГ-независимую) [1]. ПТГ-зависимая гиперкальциемия развивается при ПГПТ, третичном гиперпаратиреозе, семейной гипокальциурической гиперкальциемии [6]. ПТГ-независимая гиперкальциемия часто ассоциирована с онкологическими, гематологическими заболеваниями. Считается, что причиной повышения уровня кальция в этих случаях является синтез ПТГ-подобного пептида либо усиление остеокластической резорбции кости (остеолиз) [7].

Гранулематозные заболевания занимают особое место среди причин ПТГ-независимой гиперкальциемии. Они могут сопровождаться нерегулируемой экспрессией 1α-гидроксилазы активированных макрофагов гранулемы, что ведет к ускоренному внепочечному преобразованию 25-гидроксивитамина D в 1,25-дигидроксивитамин D. Саркоидоз и туберкулез чаще всего вызывают нарушение регуляции продукции макрофагами гена CYP27B1, кодирующего этот фермент [2].

Крайне редким вариантом является сочетание ПТГ-зависимой и ПТГ-независимой гиперкальциемии. В литературе описаны немногочисленные случаи сочетания ПГПТ и саркоидоза [8][9]. Сочетанная ПТГ-зависимая и ПТГ-независимая гиперкальциемия в виде ПТПГ и саркоидоза представлена в обсуждаемом клиническом случае.

## ОПИСАНИЕ СЛУЧАЯ

Пациентка Д., 74 года, с диагнозом «Сахарный диабет 2 типа» (СД2) в экстренном порядке была доставлена в учреждение здравоохранения «10-я городская клиническая больница» г. Минска в июле 2023 г. с жалобами на слабость, тошноту, сухость во рту. При поступлении выявлена гликемия 26 ммоль/л. Давность заболевания составила более 25 лет. Первые годы пациентка принимала таблетированные глюкозоснижающие препараты — метформин и гликлазид. Последние 10 лет была прописана инсулинотерапия инсулином короткого действия перед основными приемами пищи и инсулином длительного действия утром и перед сном в суммарной дозе 66–70 единиц в сутки. Пациентка проживала в одиночестве, поликлинику посещала нечасто, привержена к лечению не была, самоконтроль проводила нерегулярно. Из анамнеза стало известно, что у нее была ишемическая болезнь сердца со стентированием коронарных артерий, артериальная гипертензия, дислипидемия. Впоследствии к анамнезу было добавлено, что год назад пациентка стала худеть, на шее в надключичной области появилось множество узловых образований, по поводу чего она обследовалась в онкологическом центре. Со слов пациентки, окончательный диагноз выставлен не был. Пациентка была в сознании, несколько заторможена, энцефалопатична, имела клинические признаки дегидратации. У нее отмечалась сухость слизистой полости рта и языка. ИМТ — 25,5 кг/м². Деформаций позвоночника и других костных аномалий выявлено не было, указаний на переломы костей также не было. При пальпации щитовидной железы (ЩЖ) определялись верхушки обеих долей и перешеек умеренной плотности, в надключичных и подмышечных областях единичные мягко-эластичные образования (лимфоузлы). Аускультативно над легкими выслушивалось везикулярное дыхание. АД — 125/80 мм рт.ст., ЧСС — 90 в минуту. Тоны сердца были ритмичные, приглушены. Периферические отеки отсутствовали. Выявлена ацетонурия +, основные показатели кислотно-основного состояния находились в пределах референсного интервала.

Лабораторные обследования выявили повышенный уровень мочевины — 34,5 ммоль/л (2,8–7,2), креатинина — 238,2 мкмоль/л (рСКФ-EPI — 17 мл/мин/1,73 м²). Обращал на себя внимание повышенный уровень кальция — 3,08 ммоль/л (2,2–2,65). Кальций, скорректированный по альбумину, составил 3,12 ммоль/л, альбумин — 38 г/л (35–52). Выявлено повышение уровня ПТГ — 101,3 пг/мл (15–57). Значения других электролитов: калий — 4,7 ммоль/л (3,5–5,1), натрий — 136 ммоль/л (136–146), фосфор — 1,27 ммоль/л (0,81–1,45). Протеинурия в разовой порции мочи — 0,8 г/л. Лабораторное соотношение кальция и ПТГ было расценено как ПТГ-зависимая гиперкальциемия.

При анализе предыдущих выписок из лечебных учреждений и амбулаторной карты выявлены данные за нефропатию сочетанного генеза (диабетического, ишемического) с рСКФ-EPI 26–33 мл/мин/1,73 м². Лабораторно был определен крайне низкий уровень витамина D — 4,8 нг/мл.

Тяжесть состояния была обусловлена декомпенсацией сахарного диабета (СД) без развития диабетического кетоацидоза, а также уремией. На фоне в/венной титрации инсулина, инфузионной терапии в объеме 2000 мл в первые сутки и до 1500 мл в последовавшие 2 дня отмечено достаточно быстрое снижение уровня креатинина до 149 мкмоль/л (рСКФ-EPI 30 мл/мин/1,73 м²) и мочевины до 8,6 ммоль/л. Уровень скорректированного по альбумину кальция снизился до 2,6 ммоль/л.

УЗИ щитовидной (ЩЖ) и околощитовидных желез (ОЩЖ) патологических образований, подобных на аденомы ОЩЖ, не выявило.

Выполнена сцинтиграфия ОЩЖ — очага повышенной фиксации радиофармпрепарата (РФП) технеция Te99m MIBI в местах типичной локализации ОЩЖ также не было выявлено. Был выставлен клинический диагноз: «Первичный гиперпаратиероз, симптомная форма, без уточненной топической локализации. Тяжелый дефицит витамина D».

Пациентка была выписана с рекомендациями по лечению СД2, ИБС и артериальной гипертензии. Относительно ПГПТ было рекомендовано наблюдение в динамике, достаточный питьевой режим до двух литров жидкости в сутки, прием бисфосфонатов (ибандроновая кислота в таблетированной форме по 150 мг 1 раз в месяц), мониторинг уровня кальциемии через месяц после выписки. С учетом выраженного дефицита витамина D рекомендован прием колекальциферола в дозе 5000 МЕ ежедневно.

Спустя 3 недели пациентка по экстренным показаниям вновь была доставлена в приемное отделение учреждения здравоохранения «10-я городская клиническая больница» с жалобами на выраженную общую слабость, жажду, частое мочеиспускание, тошноту, рвоту и головокружение. Она была заторможена, адинамична. Периферических отеков не наблюдалось.

В приемном отделении больницы гликемия — 10 ммоль/л. Биохимический анализ крови выявил высокие значения креатинина — 237,5 мкмоль/л (рСКФ-EPI 17 мл/мин/1,73 м²), мочевины — 22,3 ммоль/л и крайне высокие значения общего кальция — 4,62 ммоль/л. Изменений в показателях кислотно-основного состояния выявлено не было.

С учетом тяжести состояния и лабораторных сдвигов пациентка была госпитализирована в отделение интенсивной терапии. Состояние было расценено как начало гиперкальциемического криза на фоне ПГПТ. Был определен уровень ПТГ — 329,5 пг/мл.

В течение двух суток проводилась регидратационная терапия под контролем центрального венозного давления (ЦВД) и диуреза. Исходно ЦВД было отрицательным, диурез снижен. В первые сутки объем инфузии составил 3800 мл, во вторые — 3100 мл. По мере восстановления ЦВД и диуреза в схему лечения добавили фуросемид 20 мг 2 раза в сутки внутривенно. К концу вторых суток уровень кальция снизился до 3,76 ммоль/л. Вместе с тем данный показатель по-прежнему находился в диапазоне, характерном для гиперкальциемии тяжелой степени. Уровень креатинина достиг значения 162 мкмоль/л (рСКФ-EPI — 27 мл/мин/1,73 м²). Диурез был достаточный, волемический статус скорректирован. Несмотря на улучшение лабораторных показателей, клиническое состояние пациентки ухудшилось. Стала прогрессировать мозговая симптоматика: вялость и заторможенность сменились суетливостью, некоторым возбуждением, дезориентацией, снижением критики. Проведенная компьютерная томография (КТ) головного мозга позволила исключить острое нарушение мозгового кровообращения. Описанные симптомы были расценены как проявление токсической гиперкальциемии.

Консервативная терапия не привела к нормализации кальция в сыворотке крови. С учетом новых клинических данных было принято решение о хирургическом лечении ПГПТ по экстренным показаниям.

Было проведено повторное УЗИ ЩЖ и ОЩЖ. В правой доле ЩЖ были зарегистрированы множественные узловые образования до 4 мм в диаметре, в толще левой доли выявлен изоэхогенный узел размерами 12,2*14,0*18,6 мм неоднородной структуры с кальцинатами (рис. 1).

**Figure:**
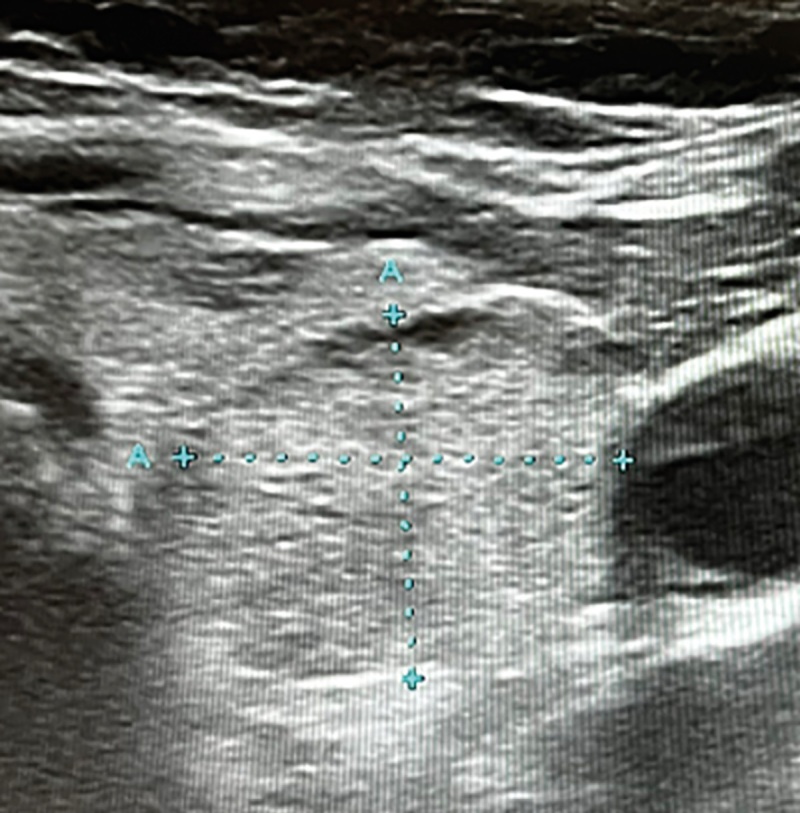
Рисунок 1. УЗИ щитовидной железы: узловое образование в толще левой доли ЩЖ размерами 12,2*14,0*18,6 мм, изоэхогенное, неоднородное, с кальцинатами.

В месте расположения нижней ОЩЖ справа выявлено гипоэхогенное образование 21,5*7,8*11,3 мм неправильной формы, неоднородной структуры, похожее на аденому ОЩЖ (рис. 2).

**Figure:**
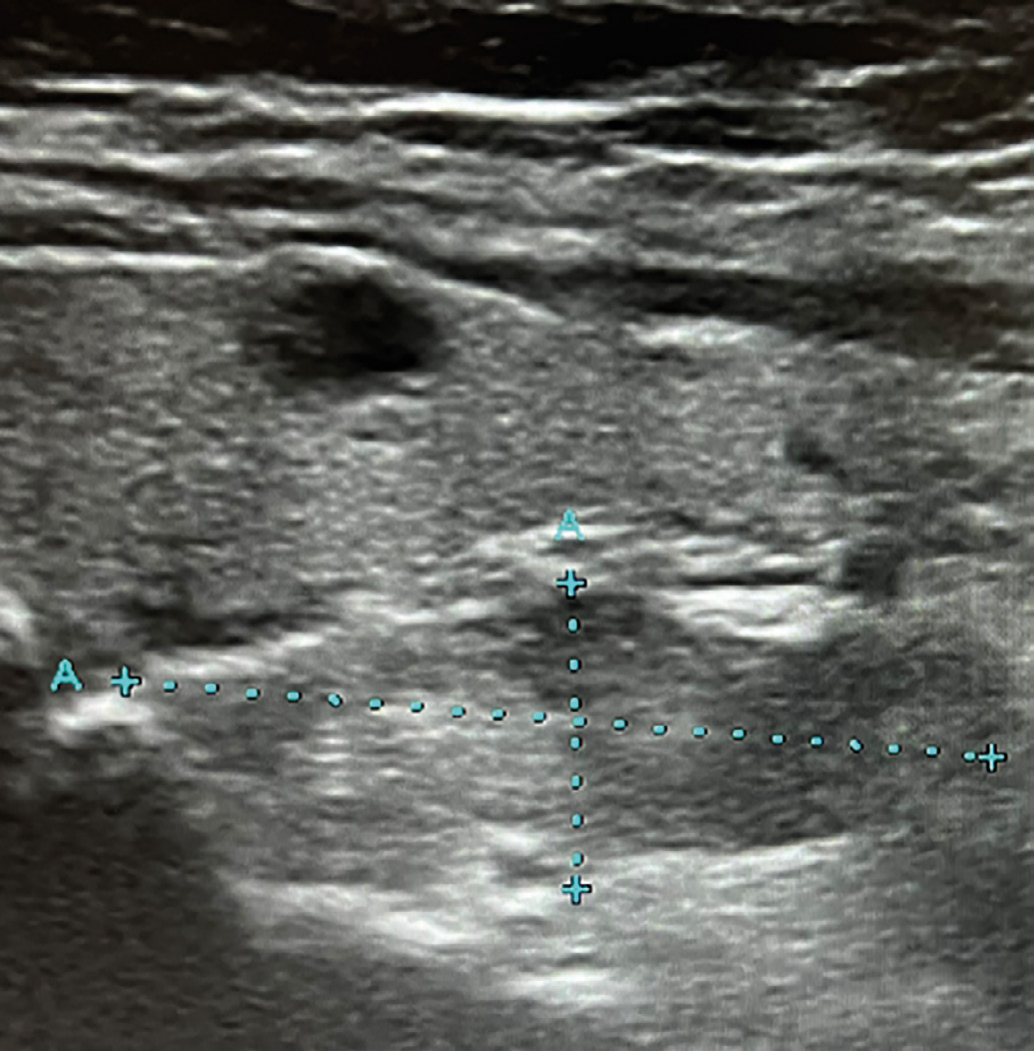
Рисунок 2. УЗИ околощитовидных желез: образование в месте локализации правой ОЩЖ размерами 21,5*7,8*11,3 мм, неправильной формы, неоднородной структуры, гипоэхогенное.

Обращало на себя внимание наличие с обеих сторон в надключичных областях гипоэхогенных образований неправильной формы дольчатой структуры — вторично измененные лимфатические узлы (рис. 3).

**Figure:**
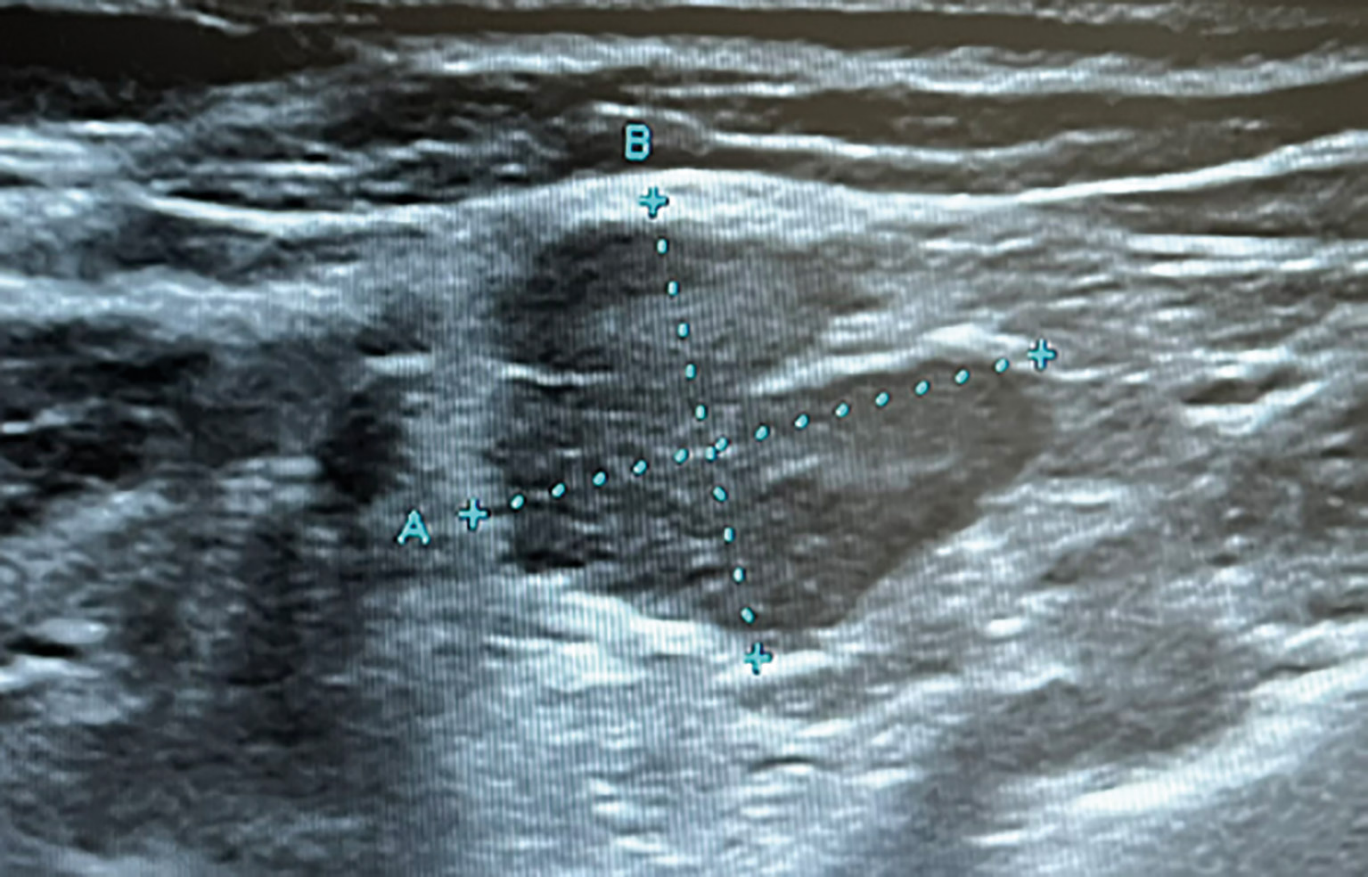
Рисунок 3. УЗИ лимфоузлов шеи: лимфоузел в надключичной области размером 14,5*14,1 мм неправильной формы, гипоэхогенный, дольчатой структуры.

Данные КТ органов грудной клетки, шеи и загрудинного пространства подтвердили наличие узлового образования в левой доле ЩЖ. Изменения в месте локализации правой ОЩЖ были неопределенными. Выявлено множество медиастинальных лимфоузлов: парааортально, паратрахеально, перикардиально, а также над- и подключично, подмышечно.

После получения данных УЗИ и КТ с описанием лимфатических узлов для уточнения анамнеза пригласили дочь пациентки. Она предоставила результаты ряда обследований (КТ органов грудной и брюшной полости, МРТ головы, ПЭТ КТ, колоноскопии, фиброгастродуоденоскопии), согласно которым данных за онкологический процесс выявлено не было. Тогда же, год назад, была проведена трепан-биопсия шейного лимфоузла, по результатам которой были обнаружены фрагменты ткани лимфоузла с нарушенной структурой за счет сливающихся мелких гранулем саркоидного типа, в отдельных полях зрения — отложения аморфного вещества по типу амилоида. Была консультирована фтизиатром — данных за туберкулез не было выявлено. Диагноз онколога: «Лимфопролиферативное заболевание с поражением лимфоузлов выше диафрагмы? Амилоидоз лимфатических узлов средостения?» Уровень кальция в сыворотке крови на тот момент повышен не был.

Согласно данным амбулаторной карты, уровень СОЭ у пациентки был повышен всегда. В настоящую госпитализацию значение СОЭ также оставалось высоким — 60 мм/час.

В ходе интраоперационной ревизии паратрахеально справа в проекции нижнего полюса правой доли ЩЖ определили округлое образование мягкой эластичной консистенции, размерами 10*20 мм, прилежащее к ЩЖ. Данное образование было удалено. Оперативное вмешательство сопровождалось лабораторным мониторингом уровня ПТГ в сыворотке крови. Забор крови на исследование был выполнен через 15 минут после удаления образования в месте расположения правой ОЩЖ. Исходный уровень ПТГ до нарушения целостности кожных покровов составлял 330 пг/мл, после удаления предполагаемой аденомы ОЩЖ содержание ПТГ в сыворотке крови снизилось незначительно и составило 286 пг/мл. По этим лабораторным данным невозможно было исключить интратиреоидное расположение еще одной аденомы ОЩЖ. Было принято решение об удалении левой доли ЩЖ, в толще которой по результатам УЗИ находилось узловое образование. После проведенной манипуляции уровень ПТГ снизился сначала до 145 пг/мл, затем до 88 пг/мл.

Динамика ПТГ во время операции и в первые сутки после операции представлена на рисунке 4.

**Figure:**
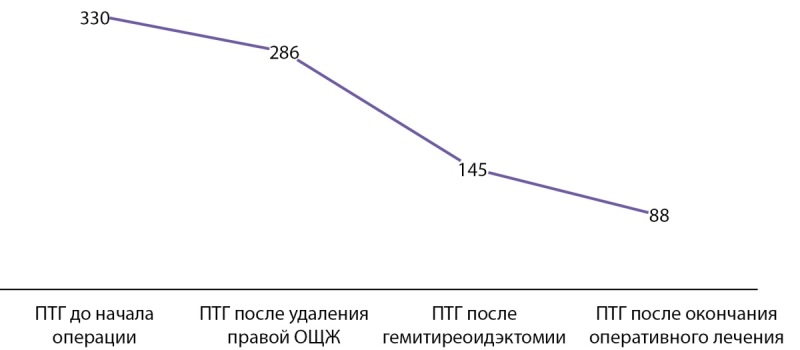
Рисунок 4. Динамика ПТГ в сыворотке крови, пг/мл.

После проведенного хирургического лечения уровень ПТГ продолжал снижаться. Динамика снижения ПТГ в послеоперационном периоде представлена на рисунке 5.

**Figure:**
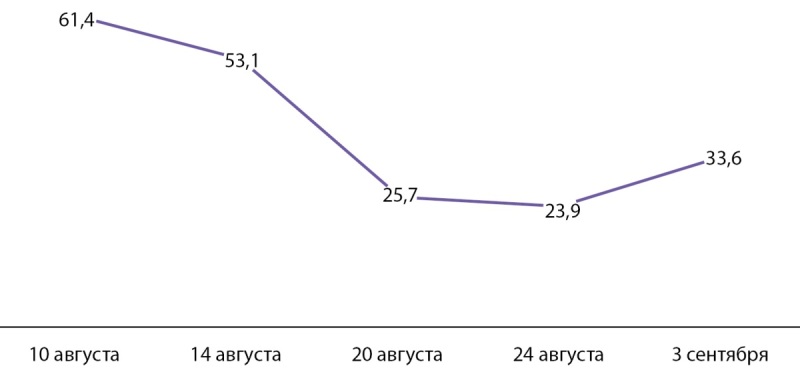
Рисунок 5. Динамика ПТГ в послеоперационном периоде, пг/мл.

К вечеру после хирургического лечения уровень кальция в сыворотке крови снизился до 2,36 ммоль/л. На следующее утро и в течение всего дня после операции уровень кальция держался на уровне 2,43–2,56 ммоль/л. Через 10 дней после хирургического вмешательства величина кальциемии стала устойчиво выше 3 ммоль/л с неуклонным нарастанием до 3,96 ммоль/л при одновременном снижении уровня ПТГ.

Проведенное гистологическое исследование подтвердило наличие аденомы ОЩЖ в образовании, прилежащем к ЩЖ справа. В удаленной левой доле ЩЖ был выявлен микро- и макрофолликулярный коллоидный зоб.

В послеоперационном периоде восстановился психоэмоциональный статус пациентки. Вместе с тем она продолжала предъявлять жалобы на слабость, потливость. Ей было тяжело дышать, особенно по ночам. Регистрировалась субфебрильная температура тела. Пневмония и ТЭЛА были исключены по данным КТ. На первый план отчетливо выступала лимфаденопатия неустановленного генеза.

Несмотря на проводимую инфузионную терапию, продолжался рост кальциемии. В связи с отсутствием в аптечной сети клиники цинакальцета и деносумаба по решению врачебной комиссии для снижения уровня кальция в сыворотке крови были назначены бисфосфонаты. Поскольку значения СКФ колебались в пределах 27–32 мл/мин/1,73 м², согласно предписаниям официальной инструкции, была введена скорректированная доза золендроновой кислоты — 2 мг в/в капельно. Введение препарата не приостановило прогрессирование гиперкальциемии. Динамика кальциемии представлена на рисунке 6.

**Figure:**
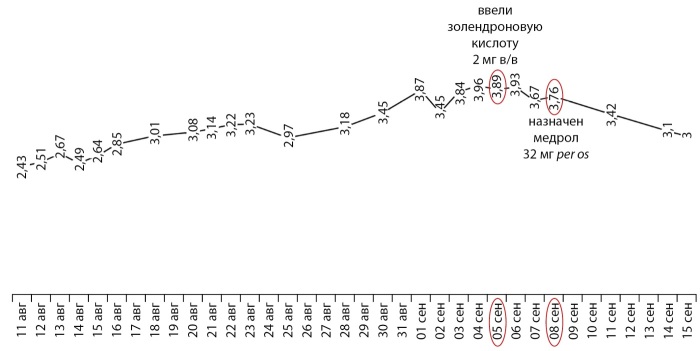
Рисунок 6. Динамика кальциемии, ммоль/л.

Было высказано предположение о наличии у пациентки саркоидоза, организовано проведение консилиума с участием врачей-пульмонологов и врачей-фтизиатров. После тщательного анализа клинических и рентгенологических данных, а также данных биопсийного материала шейных лимфоузлов (рис. 7) консилиумом пульмонологов был установлен диагноз: «Саркоидоз с поражением медиастинальных, шейных, подмышечных лимфоузлов, подтвержденный биопсией лимфоузла, активная стадия, прогрессирующее течение».

**Figure:**
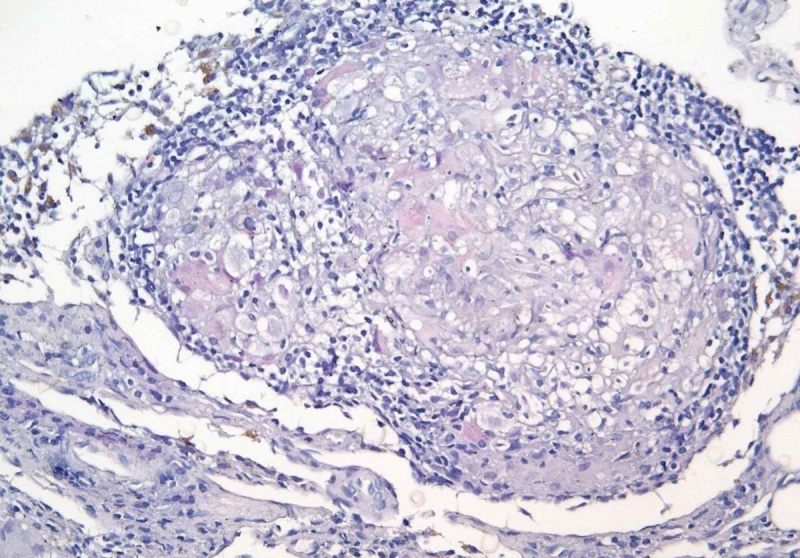
Рисунок 7. Фрагмент ткани лимфоузла с нарушенной гистоархитектурой за счет сливающихся мелких гранулем саркоидного типа. Окраска гематоксилин-эозин, ×200.

Пациентке была назначена терапия метилпреднизолоном в дозе 32 мг утром ежедневно. Спустя сутки после начала приема метилпреднизолона уровень кальция постепенно начал снижаться. Стало улучшаться клиническое состояние: уменьшилась слабость, нормализовалась температура тела, появился аппетит, пациентка перестала задыхаться по ночам. Было зафиксировано снижение уровня СОЭ до 25 мм/час. Рекомендовали продолжить терапию метилпреднизолоном в амбулаторных условиях под наблюдением пульмонолога с постепенным уменьшением дозы до полной отмены.

## ОБСУЖДЕНИЕ

В представленном клиническом случае токсическая гиперкальциемия дебютировала у коморбидного пациента с длительно существующей нефропатией сочетанного генеза, с впервые выявленным ПГПТ, а также не диагностированным ранее саркоидозом в активной стадии и дефицитом витамина D. Наличие тяжелой гиперкальциемии при значимом повышении уровня ПТГ в сыворотке крови соответствовало диагностическим критериям ПГПТ.

Согласно существующим рекомендациям, паратиреоидэктомия (ПТЭ) является единственным радикальным и эффективным методом лечения ПГПТ [6][10][11]. Однако хирургическое лечение выявленного ПГПТ в нашем клиническом случае исходно не рассматривалось. При принятии решения о проведении консервативной терапии учитывали возраст пациентки, наличие СД2 без оптимальной метаболической компенсации, ИБС со стентированием коронарных артерий в анамнезе и постоянной формой фибрилляции предсердий. Немаловажным обстоятельством стало быстрое снижение значений кальциемии до отметки менее 3 ммоль/л, а также отсутствие выявленного очага гиперпродукции ПТГ по данным УЗИ и сцинтиграфии ОЩЖ. Пожилой возраст и тяжелые соматические заболевания рассматриваются как относительным противопоказанием к хирургическому лечению [6][10][11]. Пациентке был рекомендован достаточный питьевой режим и прием бисфосфонатов в таблетированной форме.

Следует отметить, что у пациентки был выявлен тяжелый дефицит витамина D, что согласуется с данными литературы, согласно которым распространенность дефицита витамина D среди пациентов с ПГПТ выше (более 80%), чем в общей популяции (около 60%) [12]. В литературе обсуждается возможный механизм ускоренного превращения 25-гидроксивитамина D в 1,25-дигидроксивитамин D под воздействием высоких концентраций ПТГ [12]. Есть данные, что низкий уровень витамина D при ПГПТ ассоциирован с более высокими значениями ПТГ и, как следствие, более высоким содержанием кальция в сыворотке крови [12].

Для коррекции тяжелого дефицита витамина D пациентке был назначен колекальциферол в дозе 5000 МЕ ежедневно.

Причиной повторной госпитализации пациентки спустя короткое время после выписки из стационара явилась токсическая гиперкальциемия с развитием гиперкальциемического криза на фоне подтвержденного ПГПТ. Известно, что в пожилом возрасте возможны нарушения функционирования центра жажды и соответствующие несоблюдения пациентами питьевого режима, особенно в период жаркой погоды, что, вероятнее всего, и имело место в представленном клиническом случае. Терапией первой линии данного грозного осложнения при гиперкальциемии любого генеза является регидратация [13].

Проведенная коррекция волемического статуса нашей пациентки позволила снизить уровень кальция с 4,62 ммоль/л при поступлении до 3,76 ммоль/л в процессе лечения. Несмотря на проводимую терапию и некоторое снижение кальциемии, у пациентки начала прогрессировать мозговая симптоматика. Пациентка становилась все более неадекватной, перестала ориентироваться во времени и пространстве.

При развитии гиперкальциемического криза ПГПТ наиболее оптимальным способом коррекции тяжелой гиперкальциемии является удаление очага гиперпродукции ПТГ [10][11]. Возможность визуализировать аденому или несколько аденом ОЩЖ зависит от многих факторов: разрешающей способности ультразвуковых аппаратов, квалификации специалиста, особенностей эхогенной структуры и формы самого образования [6]. В обсуждаемом клиническом случае исходно УЗИ ОЩЖ не выявило патологических образований в местах локализации ОЩЖ. Сцинтиграфия ОЩЖ также не обнаружила очагов гиперфиксации РФП в областях типичного и атипичного расположения ОЩЖ.

При отсутствии визуализации образований в области шеи по данным УЗИ и сцинтиграфии ОЩЖ целесообразно выполнение КТ органов загрудинного пространства для исключения атипичного расположения аденом ОЩЖ [5][10][11] . В данном случае КТ органов грудной клетки и загрудинного пространства не позволила однозначно исключить загрудинную локализацию ОЩЖ, чтобы ограничить поле поиска очагов гиперпродукции ПТГ областью шеи. Этому препятствовали конгломераты лимфоузлов на шее и в загрудинном пространстве. Тщательное повторное УЗИ ЩЖ и ОЩЖ позволило выявить образование, подобное аденоме, в области расположения нижней правой ОЩЖ. Вместе с тем по данным УЗИ нельзя было исключить наличие аденомы ОЩЖ в толще левой доли ЩЖ.

Интраоперационная ревизия возможных мест расположения ОЩЖ рассматривается как важная опция в топической диагностике и лечении. Для оценки адекватности проведения хирургического лечения пациентам с ПГПТ рекомендуют интраоперационное определение ПТГ сыворотки крови до и через 15 минут после удаления образования [6]. Согласно проведенным исследованиям, снижение ПТГ в сыворотке крови на 50% отмечается через 15–20 минут [14]. При поражении почек период полувыведения ПТГ может увеличиваться и иметь свои индивидуальные колебания. Такая ситуация, по-видимому, имела место и в нашем случае.

В сыворотке крови, забранной через 15 мин после удаления нижней правой ОЩЖ, снижение ПТГ оказалось незначительным. Учитывая неопределенные данные УЗИ ОЩЖ, а также отсутствие снижения ПТГ после первого этапа операции, было принято решение о гемитиреоидэктомии левой доли с возможным интратиреоидным расположением еще одной аденомы ОЩЖ.

В послеоперационном периоде был продолжен мониторинг уровня кальция и ПТГ в сыворотке крови. Первоначальное снижение кальциемии до 2,36 ммоль/л сменилось постепенным подъемом ее уровня. Значения кальциемии, соответствующие токсическим, были зарегистрированы уже через 10 дней после операции. Между тем величины ПТГ в сыворотке крови продолжили постепенно снижаться и достигли средней отметки референсного интервала.

На 3–5-й день ожидалось развитие синдрома «голодных костей», представляющего собой выраженную и длительную гипокальциемию, которая возникает после ПТЭ у пациентов с тяжелым ПГПТ. Считается, что тяжелая гипокальциемия, как правило, обусловлена повышенным поступлением кальция в костную ткань вследствие внезапного прекращения влияния высоких концентраций циркулирующего ПТГ на процессы резорбции костной ткани [15]. Риск развития синдрома «голодных костей» особенно ожидаем при исходно очень высоких значениях кальциемии, как в приведенном случае [14][15][16]. В этой связи вопрос об использовании антирезорбтивных средств, таких как бисфосфонаты, для снижения острой кальциемии при ПГПТ перед операцией является актуальным. Представленные в литературе данные ограничены ретроспективными исследованиями и описаниями серий клинических случаев, результаты которых носят противоположный характер. В ряде публикаций показана протективная роль бисфосфонатов на развитие синдрома «голодных костей» [16][17], в других, напротив, сообщается об усугублении гипокальциемии после использования антирезорбтивных препаратов вследствие снижения резорбции кости, что препятствует компенсаторному выходу кальция в кровоток [18]. Последнее обстоятельство, наряду с функцией почек, является важным аргументом при принятии решения о назначении или неназначении бисфосфонатов для снижения кальциемии при ПГПТ в случаях развития гиперкальциемического криза в качестве предоперационной подготовки у пациентов с факторами риска развития синдрома «голодных костей». Было принято решение не использовать золендроновую кислоту в данной ситуации. Роль бисфосфонатов в терапии токсической гиперкальциемии при ПГПТ неоднозначна. Следует помнить о том, что их действие является отсроченным. Препараты начинают действовать через 24–48 часов [2][19].

Необъяснимый рост кальциемии у нашей пациентки в послеоперационном периоде при неуклонном снижении уровня ПТГ определил необходимость поиска других возможных причин гиперкальциемии, среди которых особое место отводилось гранулематозным заболеваниям.

В рассматриваемом клиническом случае на первый план отчетливо выступала лимфаденопатия неустановленного генеза. Было высказано предположение, что возврату токсической гиперкальциемии после успешно проведенной аденомэктомии способствовало наличие гранулематозного заболевания в виде саркоидоза. Данный диагноз был выставлен консилиумом с участием пульмонологов и фтизиатров на основании анализа представленных клинических, лабораторных, рентгенологических данных и результатов трепан-биопсии лимфоузла на шее, проведенной год назад, и недооцененной онкологом.

Как неинфекционные, так и инфекционные гранулематозные заболевания могут вызывать гиперкальциемию, опосредованную повышенной активностью 1α-гидроксилазы активированных макрофагов гранулемы. Среди них наиболее частой причиной гиперкальциемии является саркоидоз и туберкулез. По данным Gianella F. и соавт., гиперкальциемия встречается у 5,2–7,7% пациентов с саркоидозом [20].

В гомогенатах узлов при саркоидозе выявляют повышенную экспрессию гена 1α-гидроксилазы CYP27B1. Кроме того, при гранулематозных заболеваниях описаны инактивирующие мутации в гене CYP24A1, который кодирует 24-гидроксилазу, играющую важную роль в инактивации 1,25-дигидроксивитамина D и его превращении в неактивную форму 24,25-дигидроксивитамин D [21]. Сочетание повышенной экспрессии CYP27B1 и сниженной экспрессии CYP24A1 способствует избыточной продукции 1,25-дигидроксивитамина D макрофагами, что, в свою очередь, приводит к гиперкальциемии.

При бессимптомном стабильном течении саркоидоза лечение не требуется. Гиперкальциемию при саркоидозе рассматривают как проявление активности процесса, что диктует необходимость рассмотрения вопроса о назначении глюкокортикоидов. Этот класс препаратов используют при развитии среднетяжелой и тяжелой гиперкальциемии. Назначают преднизолон в дозе 40 мг или метилпреднизолон 32 мг ежедневно в течение 4-х недель с последующим снижением дозы под контролем уровня кальция в сыворотке крови [22][23]. В приведенном клиническом случае назначение метилпреднизолона привело к регрессу гиперкальциемии, снижению СОЭ и значительному улучшению общего состояния пациентки.

## ЗАКЛЮЧЕНИЕ

В приведенном клиническом случае анализ клинических, лабораторных и инструментальных данных позволил выявить редкое сочетание ПТГ-зависимой и ПТГ-независимой форм гиперкальциемии. У пациентки Д., 74 года, был диагностирован ПГПТ и саркоидоз с поражением медиастинальных и шейных лимфатических узлов. Обсуждаемый случай демонстрирует возможные сложности ведения пациентов с впервые выявленной гиперкальциемией. Пациенты с верифицированным диагнозом ПГПТ в случае принятия решения о консервативной тактике ведения нуждаются в динамическом наблюдении и контроле параметров кальциемии. Сохранение гиперкальциемии после хирургического лечения определяет целесообразность исключения как полигландулярного варианта ПГПТ, так и иных причин.

## ДОПОЛНИТЕЛЬНАЯ ИНФОРМАЦИЯ

Источники финансирования. Работа выполнена по инициативе авторов без привлечения финансирования.

Конфликт интересов. Авторы декларируют отсутствие явных и потенциальных конфликтов интересов, связанных с содержанием настоящей статьи.

Участие авторов. Все авторы одобрили финальную версию статьи перед публикацией, выразили согласие нести ответственность за все аспекты работы, подразумевающую надлежащее изучение и решение вопросов, связанных с точностью или добросовестностью любой части работы.

Согласие пациента. Пациентка добровольно подписала информированное согласие на публикацию персональной медицинской информации в обезличенной форме в журнале «Проблемы эндокринологии».
